# Pental, the New Anæsthetic

**Published:** 1892-04

**Authors:** O. N. Heise

**Affiliations:** Cincinnati, O.


### Pental, the New Anæsthetic.

BY O. N. HEISE, D. D. S., CINCINNATI, O.

Read before the Mississippi Valley Dental Association, March 11, 1892.

Called a new anæsthetic, is, after all, only a new name applied by Prof. J. V. Mering to Tri-methyl-Ethylene, having the chemical formula of $C_5H_{10}$, and is a tertiary amylene obtained by heating amylene hydrate in the presence of acids.

Pental, the present amylene, was made upon the suggestion of Prof. V. Mering by the chemical firm of C. A. Kahlbaum, in Berlin. It is a colorless liquid, of a low specific gravity [0.6783], with a boiling point of 38° [100.4] insoluble in water, but miscible in all proportions with alcohol, ether and chloroform, burns with a luminous flame, and is highly inflammable, is easily inhaled without irritating the mucous membrane of the mouth and respiratory passages in the least, [being very volatile, like chloride of Ethyl it often forms small crystals of ice on the inhaling mask, but is not decomposed on exposure to light or air.] It was used to some extent during the year of 1856-57. After 1857 we have little or nothing of note to add in regard to its use as an anæsthetic; only Pfefferman refers to it in his book of 1862, entitled, "Eine Fassliche Darstelling der gesammten Zahnheilkunde." (Comprehensive Treatise of Dental Science.) In it he makes the statement, "that he has given it some thousand times," without however mentioning how and where he obtained it. Neither describing its peculiarities as an anæsthetic, or giving any of his personal experience with its use. Since there is no further notice of it to be found in any of the medical or scientific journals after the year of 1858, it is inferred that for want of a good definite chemical article, the use of it was discontinued.

Pental was first made by Cahours, by distilling amyl-alcohol with zinc chloride, which, however, yields an indifferent, indefinite compound Amylene, containing a number of foreign substances, contaminated besides with amyl-alcohol, all these products evolve dangerous suffocating vapors, with a boiling point varying from 28°C to 75°C, besides having a very bad odor, resembling that of decaying cabbage, (or cat urine). It was first described by Ballard, in the year 1844; while Snow was the first to mention its anæsthetic properties, in 1856, for the extraction of teeth, and it is a credit, for which we may well be proud, to the dental profession, that one of its members should be the first to apply it for relief of pain in the extraction of teeth. Such was not only the case with the oldest anæsthetic, namely, Nitrous Oxide, but with several of the succeeding ones, as Ether, Bromide of Ethyl, Aethyl Chloride and others, and now Pental is brought to our notice through the same source.

It not only having been first used an an anæsthetic in dental operations by Snow, in 1856, but in being again brought to the notice of the profession for that purpose, by a man foremost in our ranks, viz.: Prof. Hollænder of Halle, who is quite enthusiastic in regard to its use, and from whose published reports of the same in the "Therapeutische Monatschrift" I have taken this meagre resume. As an anæsthetic for minor operations, as well as more prolonged ones, he not only considers it quite safe, but surpassing all others. For dental operations it not only equals Nitrous Oxide in its rapidity of action and safety, but goes ahead of it in its more prolonged action, enabling us to take more time in the performance of operations, having no unpleasant side or after effects.

Narcosis is produced in a gradual way, without any untoward symptoms, the pulse rate being slightly increased in the very beginning, but soon returning to its normal state again, and having no effect on respiration. Corneal reflex being retained for a considerable time. In some cases, whether they inhale the Pental with the eyes open or shut, the pupils are either contracted or dilated, and stare at the operator with wide open eyes, without, however, being conscious or sensible to pain; and if narcosis is not too deep will open their mouths, when shut, if commanded to do so. Spasmodic contraction of muscles, as those of mastication, as observed with Nitrous Oxide or Bromide of Ethyl, are rarely if ever noticed, and if so, will soon yield by delaying the operation for a few moments. The regaining of consciousness is as gradual as is the induction of narcosis. During the anæsthesia nor after, is there any nausea, oppression of the chest or headache, but the feeling of well-being is something that astonishes us. In fact, it is related by Prof. Hollænder, "that patients who were not feeling well, or suffering with headache, and for that reason wanted to defer taking the Pental, declared after taking it that they were feeling much better, and the headache had disappeared."

The stage of exhiliration as noticed in other anæsthetics is very seldom present, but if so, is of such a pleasant nature as to induce laughter, and Hollænder states that "it could well be called Laughing Ether."

Patients can be kept under its effects for some time without any evil consequences; but for operations in the mouth and nose it must necessarily be discontinued as soon as we begin operating; and as the sitting posture is the usual one for these operations, it is not wise or prudent to unnecessarily prolong the condition. Its effects do not seem to be lessened by frequently repeated administrations, but to the contrary, the second one is more easily induced. In the main, it may be said to differ from Chloroform, in that it acts more promptly, has no evil side or after effects. It differs from Bromide of Ethyl in that it is somewhat slower in its action, but much more lasting in its effects, and can be prolonged as it may be deemed necessary. It differs from Nitrous Oxide, in that its effects are more prolonged, and can be kept up for some time, being free from any unpleasant effects, and safe in every respect, its safety no doubt, being due to the fact of its being free from any of the halogens.

Pental, after being taken up by the blood, is probably split up into carbonic acid gas, and $H_2O$, two substances (as Hollænder puts it) which can in no wise act harmfully. One of its objections might be its peculiar odor, resembling that of oil of mustard, but being so volatile, it is soon dissipated and of little consequence.

The main drawback to its general employment will be its present high price, ($6.00 for one Kilo, 2 lbs. 8 oz.,) but with the modified Junker's Inhaler, it is stated that one Kilo, (2 lbs. 8 oz.,) is enough for about fifty administrations; some of them being more or less prolonged.

Method of administration about the same as that for Ether, or better by means of a slightly modified Junker's Inhaler. Pental being so volatile that if the ordinary methods are pursued, it takes much longer to induce the narcotic condition, for the reason that too much of it is lost, also making it more expensive. For short minor operations, as the extraction of two to four teeth, it is not necessary to bring about deep narcosis, as it produces insensibility in its very first stage. Its full anæsthetic effects are brought about in from three to five minutes, lasting four to five minutes.

As to the initial signs of Pental Anæsthesia, there are really none to be spoken of as being definite and pronounced, as there is hardly any change of expression, except the occasional stare of the wide open eyes, the color of the face remaining the same, Corneal reflex being retained for some time. But the indifference of the patient, with the relaxation of the muscles, and the apparent resemblance to the normal sleep, are a sufficient guide to show that the patient is under its effects, and that we can safely begin with the operation without hurrying ourselves as we are obliged to do with Nitrous Oxide.

Prof. Hoællnder sums up his experience with Pental as follows: "That we have in it one of the safest, surest and most pleasant anæsthetics as yet brought before the profession." Surpassing all others in his estimation, having been given many hundreds of times, even by persons not familiar with its action or influence on the system, without producing any evil consequences, and always acting satisfactorily, having had no effect on the system in regard to the production of sugar in the urine, he having paid especial attention to that point.

In conclusion, I would state, that just in proportion as we treat the mouth and teeth from a medical standpoint, and get away from the mere mechanical idea of Dentistry, and look upon the mouth as a whole, and not as composed of so many individual teeth, treating and filling a few of them and leaving the balance of the mouth in a deplorable condition, which, I am sorry to say, is so frequently done, (even by men in the front ranks of our profession) and as we come to consider the adjacent parts as rightfully belonging to our specialty, just in that proportion will we make use of anæsthetics more and more; and what a blessing to suffering humanity if the favorable reports in regard to Pental, by such eminent men as Professors Hollænder and J. V. Mering should be confirmed by others.

---

DISCUSSION OF DR. HEISE'S PAPER ON PENTAL.

*Dr. Cassidy.*—I sincerely hope this new anæsthetic, Pental, will prove a safer and in every way a better one than those we already know. It is certainly an improvement in point of color, at least, on what was known as amylene, which was used for the same purpose some forty years ago.

According to Dr. Heise's excellent paper the formula of

Pental is $C_5H_{10}$, it is therefore isomeric with amylene $C_5H_{10}$ or Isopentane; isologous with pentane $C_5H_{12}$ and homologous with ethylene $C_2H_4$, the latter also known as heavy carburetted hydrogen. All these hydrocarbons are, however, regarded as being made up of groups of radicles, each radicle having its own special position in the molecule; so that compounds isomeric with each other, having the same kind and number of atoms, will differ in physical and perhaps more or less in chemical properties. For instance, amylene was prepared by the dehydrating influence of zinc chloride on amyl alcohol, or fusel oil $C_5H_{12}O$, and Pental it seems is obtained by the action of acids on amylene hydrate. Now, amyl alcohol and amylene hydrate have the same rude formula $C_5H_{12}O$, but if some of the radicles of which they are composed be placed by the simplest change possible we recognize two entirely different bodies: $C_5H_{11}$ [OH] amyl alcohol $C_5H_{10}$ [CHOH] amylene hydrate.

If these two alcohols be acted on by the same dehydrating agent, as either zinc chlorid, sulphuric acid, or phosphoric oxide, it is probable that a different product would in each case result. Nevertheless, I believe that pure amylene and pure pental are one and the same.

*Dr. Heise.*—How about the boiling point of amylene varying from 32° to 78°, while the boiling point of pental is constant at 38°?

*Dr. Cassidy.*—That is due solely to the purity of pental. Pure amylene has, of necessity, a constant boiling point which is doubtless also 38°. The homologous liquid hydrocarbons increase in their boiling point by about 30° for every addition of $CH_2$, consequently if there be more or less of other closely related hydrocarbons present with the one experimented with, which is generally the case, as for instance: butylene $C_4H_8$, and hexylene $C_6H_{12}$, which are immediately above and below amylene respectively in the homologous series, I think I am justified in saying that such a mixture will accordingly vary in its boiling point. But any pure liquid free from every admixture has, as is well known, its definite boiling point as well as its definite vapor density.

The question alluded to in the paper as to the probable decomposition of pental in the animal body into $CO_2$ and $H_2O$, it seems is not fully settled, but may be by careful examination of the excretions. My own humble opinion is that it does not so suffer chemical change, but on the contrary, that the action of this hydrocarbon, like those of common illuminating gas, may be more in the direction of suffocation, thus preventing the escape of the normal $CO_2$, and the consequent increase of the latter, the natural anæsthetic within the body, thereby obtunding the nerve centres and lessening the conductivity of the sensory nerves. However, if pental does undergo decomposition in the body, as stated, the $CO_2$ thus formed would be retained for the time being and induce insensibility to pain much like tying a rope around a man's neck for the same purpose.

I am satisfied that any process which will develop in the body an increased quantity, or retention of $CO_2$ as by rapid breathing, unusual excitement, or violent exercise, like playing base ball or running to a fire, will at the same time reduce our insensibility to pain. So also with $N_2O$ which evidently acts by superseding the iron compound in carrying O to the tissues and at the same time causing therein a larger amount than usual of $CO_2$ to be formed. The retention of the latter, the natural anæsthetic, on account of its inability to find the normal reduced iron oxide, and thus escape the usual way, as ferrous carbonate through the venous circulation, seems to me to be the ultimate cause of the anæsthesia rather than by the immediate influence of the $N_2O$.

It is not finding fault with pental to assume that it does not undergo decomposition when inhaled, in fact it is otherwise; for if chemical changes take place by the union of its ıadicals with radicles other than O, there is no telling what complications might result under certain conditions.

The paper states that pental is formulated as tri-methyl ethylene $CH_3\ CH_3 = C = CH—CH_3$.

This is, of course, the possible arrangement of the radicles in each molecule, which may be decided later on by stereo-chemistry; but with all respect to the essayist I will say, that taking the peculiar odor of pental into the question might it not be more truthfully and simply formulated as consisting of the direct union of the two hydro-carbon radicles allyl $C_3H_5$ and ethyl $C_2H_5 = C_3H_5—C_2H_5$, inasmuch as allyl is the characteristic hydro-carbon radicle of mustard oil.

I wish to congratulate Dr. Heise for thus introducing this new anæsthetic to us, and say that on account of its high price it will not be, at least for the present, within the reach of advertising dentists.

*Dr. J. Taft.*—Is there any probability of the cost in its manufacture being reduced.

*Dr. Cassidy.*—I do not see why it should not be made at greatly cheaper rates. The olefines can be formed in various ways; by abstraction of the elements of water, by dehydrating agents from amyl alcohol, or fusel oil, which is quite cheap you know, or from its homologous aclohols, and by decomposition of petroleum compounds, which exist in illimitable quantities in mother earth. If the demand so justifies a comparatively cheap process of manufacture will doubtless be discovered.

